# Spiritual-Religious Profiles and Psychological Distress in Romanian Patients with Cancer

**DOI:** 10.3390/curroncol33100573

**Published:** 2026-09-22

**Authors:** Dana Sonia Nagy, Vlad-Norin Vornicu, Alina-Gabriela Negru, Alina Doina Tănase, Serban Mircea Negru

**Affiliations:** 1 Doctoral School, “Victor Babes” University of Medicine and Pharmacy, Eftimie Murgu Square No. 2, 300041 Timisoara, Romania; dana.nagy@umft.ro; 2 Department of Oncology, “Victor Babes” University of Medicine and Pharmacy, Bd. Victor Babes No. 16, 300226 Timisoara, Romania; vlad.vornicu@umft.ro (V.-N.V.); serban.negru@umft.ro (S.M.N.); 3 Department of Palliative Care, OncoHelp Hospital Timisoara, Ciprian Porumbescu Street No. 59, 300239 Timisoara, Romania; 4 Department of Cardiology, “Victor Babes” University of Medicine and Pharmacy, 300041 Timisoara, Romania; alinanegru@umft.ro; 5 Department of Professional Legislation in Dental Medicine, Faculty of Dental Medicine, “Victor Babes” University of Medicine and Pharmacy, Eftimie Murgu Square No. 2, 300041 Timisoara, Romania; 6 Research Centre in Dental Medicine Using Conventional and Alternative Technologies, Faculty of Dental Medicine, “Victor Babes” University of Medicine and Pharmacy, Eftimie Murgu Square No. 2, 300041 Timisoara, Romania

**Keywords:** spiritual well-being, psychological distress, cancer, latent profile analysis, religiosity, quality of life, prediction model

## Abstract

**Background/Objectives:** Religious engagement may coexist with low existential well-being. We identified spiritual-religious profiles in Romanian patients with cancer, assessed their associations with distress and quality of life, and evaluated spirituality's incremental predictive value. **Methods:** This single-centre cross-sectional study included 175 adults with cancer. Latent profile analysis used FACIT-Sp Meaning, Peace, and Faith, Religious Commitment Inventory-10, and service attendance. Clinically significant distress was defined as Hospital Anxiety and Depression Scale anxiety or depression scores ≥8. Adjusted regression and a six-predictor logistic model with bootstrap internal validation were used. **Results:** Three profiles emerged: low spiritual-religious resource (*n* = 61), religiously engaged but existentially unsettled (*n* = 52), and integrated spiritual-religious resource (*n* = 62). Distress prevalence was 68.9%, 61.5%, and 25.8%, respectively (*p* < 0.001). Compared with the low-resource profile, the integrated profile had lower adjusted odds of distress (OR 0.19, 95% CI 0.08–0.44), higher global quality of life (adjusted difference 14.1 points, 95% CI 4.4–23.8), and lower symptom burden (−11.8 points, 95% CI −18.6 to −5.0). The full model had an apparent AUC of 0.816 (95% CI 0.748–0.875) and an optimism-corrected AUC of 0.789. Its AUC exceeded that of the clinical-only model by 0.064 (95% CI 0.008–0.12; *p* = 0.023). **Conclusions:** Religious involvement alone did not indicate resolved spiritual needs. Inner peace may improve distress risk assessment, although prospective external validation is required.

## Simple Summary

Many people with cancer draw on faith and religious practice to cope, and it is often assumed that patients who are religiously active are spiritually at peace. We asked whether this is true in Romania, where religious practice is nearly universal. Among 175 adults with cancer we identified three patterns of spiritual and religious resources. About one patient in three was highly religious yet reported little inner peace or sense of meaning, and these patients were almost as likely to have clinically significant anxiety or depression as patients with few spiritual resources at all; only patients who combined religious engagement with inner peace had markedly lower distress and better quality of life. A short measure of inner peace also improved the identification of distressed patients beyond the clinical information routinely available. These findings suggest that asking patients about religious affiliation or practice is not enough, and that a brief assessment of inner peace could help clinicians recognise patients who need psychological or spiritual support.

## 1. Introduction

Cancer accounted for approximately 20 million new diagnoses and 9.7 million deaths worldwide in 2022, and the absolute burden continues to rise as populations age [1]. Europe bears a disproportionate share of this burden, and within Europe the countries of the eastern periphery, including Romania, are characterised by later stage at presentation and slower improvement in cancer mortality than the continental average [2]. A direct consequence is that a large fraction of the oncology caseload in these settings consists of patients with advanced disease for whom psychological morbidity is not an incidental complication but a defining feature of the illness experience. Structured psychiatric interviewing across major tumour entities has shown that roughly a third of patients with cancer meet criteria for a mental disorder within a four-week window, with adjustment, anxiety and mood disorders predominating [3].

Recognition of this burden has translated into explicit clinical guidance. National Comprehensive Cancer Network guidelines have long recommended that every patient be screened for distress at the initial visit and at appropriate intervals thereafter, with defined referral pathways for those who screen positive [4]. Yet meta-analytic work across oncological, haematological and palliative care settings shows that the prevalence of depression, anxiety and adjustment disorder varies substantially with setting and case-mix, and that a considerable proportion of cases go unrecognised in routine practice [5]. Screening instruments capture current symptoms efficiently but say little about which patients are vulnerable and why, and they offer no guidance on what kind of support a given patient is likely to need.

Spirituality is one of the most frequently invoked candidate resources in this context. The 2009 Consensus Conference established spiritual care as a required dimension of quality palliative care and defined the screening, history-taking and assessment framework still in use [6], and a comprehensive evidence review of spirituality in serious illness concluded that spiritual factors are meaningfully associated with patient-reported outcomes and should be addressed within routine care rather than left to chance [7]. What this literature has largely not resolved is whether the various components of spirituality—belief, religious practice, sense of meaning, inner peace—function as a single resource or as separable, and possibly discordant, ones.

This question cannot be answered by variable-centred methods. Regression tells us the average effect of a unit change in each spirituality measure across the whole sample; it cannot tell us whether there exist patients in whom high religious commitment coexists with low existential well-being, nor how such patients fare. Latent profile analysis, by contrast, can identify individual-level discordant configurations—high religious commitment alongside low inner peace—that a regression coefficient averages over and therefore overlooks, and it can estimate how many patients occupy such a configuration and how they fare; this is the principal reason for adopting a person-centred approach here. Person-centred methods such as latent class and latent profile analysis address exactly this gap by identifying unobserved subgroups whose members share a characteristic configuration across several indicators; robust simulation work has established the fit criteria by which the number of such subgroups should be chosen [8]. The approach has recently begun to be applied to spirituality in oncology, where latent profiles of spiritual well-being have been shown to relate systematically to perceived social support and hope [9] and, in advanced cancer, to psychological distress, financial burden and family function [10], but it has rarely been used to test whether religious engagement and existential well-being can come apart within the same patient.

The Romanian setting is unusually well suited to this question. Religious affiliation is near-universal and overwhelmingly Orthodox, and formal religious practice is culturally normative, so religious commitment approaches a constant while the existential dimensions of spiritual well-being retain wide interindividual variation. In a population where almost everyone is religious, any patient who is religiously engaged yet lacks inner peace becomes visible as a distinct configuration rather than being absorbed into a general low-spirituality group. If such a configuration exists and carries elevated risk, it would have immediate clinical consequences, because these are precisely the patients whom a clinician performing a brief religious history would be most likely to classify as spiritually well resourced and therefore least likely to refer for support. 

We therefore pursued two linked objectives in a consecutively recruited Romanian cancer cohort. The first was to identify latent spiritual–religious profiles from a set of existential and religious indicators, to characterise them clinically and demographically, and to quantify their association with clinically significant psychological distress and with quality of life. The second was to determine whether spirituality measures add incremental predictive information beyond routinely available clinical variables, by developing and internally validating a logistic risk model for clinically significant distress and evaluating its discrimination, calibration and clinical utility. The two objectives are sequential rather than parallel: the first asks which patients exist—whether a discordant configuration is present in this population and what burden of distress it carries—whereas the second asks how such patients can be identified in routine practice, so that together they form a chain from phenomenological discovery to clinical screening. A secondary propensity-score analysis examined whether seeking spiritual support during illness was itself associated with lower distress. 

## 2. Materials and Methods

### 2.1. Study Design, Setting and Population

This was a single-centre, cross-sectional, observational study conducted within the oncology and palliative care services of Timisoara, Romania. Consecutive adults attending for systemic anticancer therapy, surveillance or supportive and palliative care were invited to participate during routine clinical contacts between September 2024 and June 2025. Assessment was made at a single time point using a structured, interviewer-supported questionnaire administered in Romanian, with clinical data abstracted contemporaneously from the medical record. The study is reported in accordance with the STROBE recommendations for observational research.

The recruitment target was set before enrolment from the requirements of the prediction objective, which was the more demanding of the two. A logistic model with six prespecified predictors requires at least 10 outcome events per predictor to limit overfitting [11], that is, a minimum of 60 patients with clinically significant distress; at an anticipated prevalence of approximately 40% for HADS-defined distress, in line with pooled estimates for mixed oncology and palliative settings [5], this corresponded to 150 patients, inflated to 165 to allow for up to 10% incomplete questionnaires. For the profile-contrast objective, detecting a difference in distress prevalence of 30 percentage points (30% in the most favourable profile versus 60% in the remaining profiles, an odds ratio of approximately 0.29) with a 1:2 allocation, a two-sided α of 0.05 and 80% power required 96 patients (32 and 64), so the prediction objective governed the target. Recruitment continued until the target had been exceeded. The analytic sample comprised 175 patients with 90 outcome events, giving 15 events per predictor in the six-predictor final model, comfortably above the conventional minimum of 10 and therefore unlikely to produce materially overfitted coefficients. The study was approved by the Ethics Committee of the OncoHelp Association, Timisoara (approval no. 1657, 16 July 2024), was conducted according to the Declaration of Helsinki, and written informed consent was obtained from every participant. Data were pseudonymised at entry and the analysis dataset contained no direct identifiers.

### 2.2. Eligibility Criteria and Group Definition

Patients were eligible if they were aged 18 years or older, had a histologically or cytologically confirmed malignancy of any site and stage, were aware of their diagnosis, and were able to consent and complete a Romanian-language questionnaire. Exclusion criteria were documented cognitive impairment, delirium or severe psychiatric illness precluding reliable self-report, a clinical condition making participation unduly burdensome in the treating clinician’s judgement, or refusal. Patients at all disease stages were admitted, including those in remission, in order to preserve the full range of both distress and spiritual well-being.

Because this is an observational study, groups were not allocated by the investigators but derived empirically. Latent profile membership, estimated as described in Section 2.4, defined the study groups. For the adjusted comparative analyses the profile with the most favourable indicator configuration served as the index group and the remaining profiles as the comparison group, a contrast specified a priori on the grounds that the clinically actionable question is whether an integrated spiritual–religious configuration confers protection relative to any other configuration. As a distinct secondary exposure, patients were classified by whether they reported having sought spiritual support during their illness, a self-selected behaviour for which propensity-score methods were used to construct a comparable control group.

### 2.3. Variables, Outcomes and Data Collection

Five indicators were used to define the latent profiles. Three were the subscales of the 12-item Functional Assessment of Chronic Illness Therapy—Spiritual Well-Being scale [12], scored according to the validated three-factor structure as Meaning (items 2, 3, 5 and 8), Peace (items 1, 4, 6 and 7) and Faith (items 9–12), with items 4 and 8 reverse-keyed [13]. The fourth was the total score of the Religious Commitment Inventory-10, a validated 10-item measure of the behavioural and cognitive salience of religion [14]. The fifth was an ordinal measure of the frequency of attendance at religious services, ranging from rarely to daily. This combination was chosen so that the existential dimensions of spirituality and the practice-based dimensions of religiosity were represented in comparable numbers, allowing profiles to separate along either axis. All indicators were standardised to zero mean and unit variance before modelling so that no indicator dominated by virtue of its scale range.

The primary outcome was clinically significant psychological distress, defined a priori as a score of 8 or more on either subscale of the Hospital Anxiety and Depression Scale, the conventional case-finding threshold for this instrument [15]. Three alternative outcome definitions were prespecified for sensitivity analysis: anxiety alone at the same threshold, depression alone, and the more stringent threshold of 11 or more on either subscale, which identifies probable rather than possible cases. Quality of life was measured with the EORTC QLQ-C30 [16], scored by linear transformation to a 0–100 metric with higher functional and global scores denoting better status and higher symptom scores greater burden; a composite symptom burden score was defined a priori as the unweighted mean of the eight symptom scales, and a functional summary score as the mean of the five functional scales. Candidate predictors for the risk model comprised the three FACIT-Sp subscales, RCI-10 score, attendance, spiritual help-seeking, composite symptom burden, ECOG performance status, age, sex, advanced disease status, residence, comorbidity count, active chemotherapy and time since diagnosis. Internal consistency in this sample was 0.879 for the FACIT-Sp-12 total, 0.765 for Peace, 0.727 for Meaning, 0.887 for Faith, 0.931 for the RCI-10, 0.859 for HADS-anxiety and 0.778 for HADS-depression. Three HADS items recorded against the inverted response column of the local questionnaire were re-keyed to the validated scoring direction before analysis.

In response to peer review, the integrity, coding and scoring of the three psychometric instruments were verified against the original paper questionnaires before the analyses were finalised. Two investigators independently re-entered every item of the FACIT-Sp-12, the RCI-10 and the HADS for all 175 participants from the source documents, blind to the original database; item-level agreement between the two re-entries was 99.6%, and the 34 discrepant entries were resolved by joint inspection of the source form. The verification established three points. First, the four-point administration of the FACIT-Sp-12 was a property of the locally printed questionnaire, on which all 12 items carried four ordered response options rather than the validated five; it affected every participant identically, no value outside 1–4 was found, and the re-entered subscale scores reproduced the analysed scores exactly. The subscales were therefore analysed on their observed 4–16 metric. Because all profile indicators are standardised before mixture modelling, and because discrimination, calibration and net benefit are invariant to a linear rescaling of a predictor, neither the latent profile solution nor the performance of the risk model depends on this choice; the reported per-point odds ratios can be converted to the validated 0–16 metric by raising them to the power 0.75 (for FACIT-Sp Peace, 0.72 per point on the 4–16 metric corresponds to 0.78 per point on the 0–16 metric). Second, the re-entry recovered item-level data for all 10 RCI-10 items. The recomputed 10-item totals matched the analysed totals for 164 participants (93.7%); the 11 discrepant totals, which were the totals below the nominal minimum of 10 and account for the observed range of 8–46, belonged to participants who had left one or two RCI-10 items unanswered and whose total had been entered as the raw sum of the completed items. These totals were rescored by prorating, as the mean of the completed items multiplied by 10, which is appropriate when at least eight of the ten items are answered; after rescoring, all totals lay within the 10–50 range of the instrument, and no total changed by more than 2.5 points. Third, the HADS re-keying was confirmed against the source questionnaires: on the local form the response options of three items were printed in the reverse order of the validated version, so that the transcribed values for those items ran opposite to the scoring key. Keying them in the validated direction yielded positive corrected item–total correlations for all 14 items (range 0.31–0.71), whereas leaving them as transcribed produced negative item–total correlations for exactly those three items, and the re-keyed totals reproduced the analysed HADS scores exactly. Because the rescoring altered only 11 RCI-10 totals by a small amount and the FACIT-Sp metric is immaterial to the standardised indicators, the prespecified analyses reported in Section 3.1, Section 3.2, Section 3.3, Section 3.4, Section 3.5, Section 3.6, Section 3.7 and Section 3.8 are based on the verified dataset with the instruments scored as originally recorded; all key analyses were then repeated with the prorated RCI-10 totals, with the profiles re-estimated, and with the FACIT-Sp subscales rescaled to the 0–16 metric, and the results are reported alongside the other sensitivity analyses in Section 3.9.

### 2.4. Statistical Analysis

Latent profile analysis—the continuous-indicator form of latent class analysis—was performed by fitting finite Gaussian mixture models with diagonal covariance structure to the five standardised indicators, estimating solutions from one through five profiles with 100 random starts per solution and a variance floor to prevent degenerate likelihood spikes. Model selection followed established practice [8] and considered the Akaike information criterion, the Bayesian information criterion, the sample-size-adjusted Bayesian information criterion, relative entropy as a measure of classification certainty, the smallest class size, and a parametric bootstrap likelihood-ratio test comparing each solution with the solution containing one fewer profile using 200 bootstrap replicates. Patients were assigned to their modal profile, and average posterior probabilities of assignment are reported by profile as a measure of classification quality.

Descriptive comparisons across profiles used one-way analysis of variance, the Kruskal–Wallis test or Pearson’s χ^2^ test as appropriate, with η^2^ and Cramér’s V as effect sizes. Adjusted comparisons of continuous outcomes used linear regression with HC3 heteroscedasticity-consistent standard errors, and adjusted comparisons of binary outcomes used logistic regression, in both cases controlling for age, sex, advanced disease, ECOG performance status and comorbidity count. Effect modification was assessed by fitting multiplicative interaction terms in the full cohort and reporting the interaction *p* value alongside subgroup-specific odds ratios; subgroup models omitted comorbidity count to preserve stability in small strata, and where a model still failed to converge because of quasi-separation the covariate set was reduced further in a prespecified order until a stable estimate was obtained. Estimates from the subgroup models therefore differ marginally from the correspondingly adjusted estimates elsewhere.

The prediction model was developed and is reported in accordance with the TRIPOD statement for transparent reporting of a multivariable prediction model for individual prognosis or diagnosis [17], covering model development with internal validation and no external validation cohort. The risk model was a multivariable logistic regression containing six prespecified predictors chosen on clinical grounds and constrained by the available events: FACIT-Sp Peace, FACIT-Sp Faith, composite symptom burden, ECOG performance status, spiritual help-seeking and sex. No stepwise selection was used, so the reported standard errors are not inflated by data-driven variable choice. Discrimination was quantified by the area under the receiver operating characteristic curve with a 1000-resample bootstrap confidence interval; overall accuracy by the Brier score; and explained variation by Nagelkerke’s R^2^. Calibration was assessed by the calibration slope and calibration-in-the-large from a logistic recalibration model, by a decile-based calibration plot, and by the Hosmer–Lemeshow test. Optimism in discrimination, calibration slope and Brier score was estimated by the Harrell bootstrap procedure with 500 resamples, in which the model is refitted in each bootstrap sample and the difference between its apparent and test performance is averaged and subtracted from the apparent estimate [18]; calibration was interpreted as the primary determinant of clinical usefulness [19]. Two comparator models were fitted, one containing only clinical predictors and one only spiritual predictors, and their areas under the curve were compared by a 2000-resample bootstrap of the paired difference.

Clinical utility was evaluated by decision-curve analysis, in which net benefit is computed across a range of threshold probabilities as the true-positive rate minus the false-positive rate weighted by the odds of the threshold, and compared with the default strategies of treating all and treating no patients [20]. For the secondary exposure of spiritual help-seeking, a propensity score was estimated by logistic regression on age, sex, advanced disease, ECOG status, comorbidity count, residence, education and religious commitment, and used both for stabilised inverse-probability-of-treatment weighting truncated at the 1st and 99th centiles and for 1:1 nearest-neighbour matching on the logit of the propensity score within a calliper of 0.2 standard deviations, with balance assessed by standardised mean differences against a threshold of 0.10 [21]. Missing data affected no more than 4.0% of observations for any variable (time since diagnosis, seven patients; under 1% for all others) and were handled by complete-case analysis in the primary analyses. Because complete-case estimates are unbiased only if missingness is unrelated to the outcome given the observed covariates, two missing-data sensitivity analyses were added: patients with and without any missing value were compared on profile membership, distress status and the remaining analysis variables to characterise the missingness pattern, and the adjusted profile–distress models and the risk model were re-estimated in 20 datasets completed by multiple imputation by chained equations, with all analysis variables and the outcome included in the imputation model and estimates combined by Rubin’s rules. All tests were two-sided with α set at 0.05. Analyses were classified a priori as confirmatory or exploratory. The confirmatory analyses were the profile enumeration, the primary contrast between the integrated profile and the other profiles for clinically significant distress, and the comparison of the areas under the curve of the full and clinical-only models; each tests a single prespecified hypothesis and is reported at the nominal α without correction. All remaining analyses—secondary outcomes, alternative outcome definitions, subgroup, sensitivity and propensity-score analyses, and the univariable screen of candidate predictors—are exploratory. Exact *p* values are reported throughout so that any correction can be applied, and for the family of twelve outcome comparisons in Section 3.4, Holm-adjusted *p* values are additionally reported in the text. Analyses used Python 3.11 with pandas, statsmodels, scikit-learn and SciPy.

## 3. Results

### 3.1. Enumeration of Latent Spiritual–Religious Profiles

Latent profile solutions from one through five classes were fitted to the five standardised spiritual–religious indicators; the corresponding fit statistics are presented in Table 1.

The three-profile solution had the lowest BIC (2179.6; four profiles 2184.2; five profiles 2184.6) and entropy of 0.847. The three classes contained 61, 52 and 62 patients, with mean posterior assignment probabilities of 0.946, 0.915 and 0.940, respectively.

### 3.2. Characterisation of the Three Profiles

The configuration of indicator values that defines each profile, together with the illness-related spirituality variables not used in the modelling, is presented in Table 2.

All five profile-defining indicators differed across classes (all *p* < 0.001). Class 1 scored below the sample mean on all indicators; Class 2 had high religious commitment and attendance but lower Peace and Meaning; and Class 3 scored high across both existential and religious indicators. Spiritual support had been sought by 41.0%, 90.4% and 88.7% of Classes 1–3, respectively.

### 3.3. Demographic and Clinical Characteristics by Profile

Demographic and clinical characteristics across the three profiles are summarised in Table 3.

Sex (*p* = 0.007), residence (*p* = 0.025) and ECOG performance status (*p* = 0.009) differed across profiles. Age, education, marital status, tumour site, disease status, time since diagnosis, comorbidity count, chemotherapy, surgery and religious affiliation did not differ significantly.

### 3.4. Psychological Distress and Quality of Life by Profile

Unadjusted and adjusted differences in psychological distress and quality of life outcomes across the three profiles are presented in Table 4, and the underlying joint structure of inner peace and religious commitment is displayed in Figure 1.

Clinically significant distress was present in 68.9% of Class 1, 61.5% of Class 2 and 25.8% of Class 3 (*p* < 0.001). For Class 3 versus Class 1, the adjusted odds ratio was 0.19 (95% CI 0.08 to 0.44; *p* < 0.001). Class 3 also had higher global quality of life (adjusted difference 14.1 points, 95% CI 4.4 to 23.8) and lower composite symptom burden (−11.8 points, 95% CI −18.6 to −5.0). After Holm correction across the twelve adjusted comparisons in Table 4, all differences remained significant (adjusted *p* ≤ 0.012) except social functioning (adjusted *p* = 0.552).

Across each RCI-10 tertile, distress prevalence was higher in the lowest than in the highest Peace tertile. The highest observed prevalence was 95.2% (20/21) in the low-Peace/middle-RCI-10 cell, and the lowest was 7.1% (1/14) in the high-Peace/middle-RCI-10 cell.

### 3.5. Subgroup Analyses

To assess the consistency of the association between the integrated profile and clinically significant psychological distress across clinically relevant strata, prespecified subgroup analyses with interaction tests were performed (Table 5).

The adjusted odds ratio for distress for Class 3 versus Classes 1–2 was 0.22 (95% CI 0.10 to 0.44; *p* < 0.001). Subgroup estimates remained below 1 across sex, age, disease status, ECOG, residence and symptom-burden strata, and none of the interaction tests was significant (all *p* for interaction ≥ 0.347).

### 3.6. Univariable Associations with Clinically Significant Distress

Univariable associations between every candidate predictor and clinically significant distress, with the discriminative ability of each predictor considered alone, are shown in Table 6.

FACIT-Sp Peace showed the strongest univariable discrimination (OR 0.65 per point, 95% CI 0.56 to 0.76; AUC 0.784; *p* < 0.001). Composite symptom burden was also associated with distress (OR 1.56 per 10 points, 95% CI 1.30 to 1.87; AUC 0.743; *p* < 0.001). Age, sex, advanced disease, residence, comorbidity count, active chemotherapy and time since diagnosis were not significant univariable predictors.

### 3.7. Correlation Structure Among Predictors and Outcomes

The rank correlation structure among the spirituality predictors, clinical variables and outcome measures is presented in Table 7.

Peace correlated with Meaning (ρ = 0.63), Faith (ρ = 0.39) and RCI-10 (ρ = 0.26). Its correlations with HADS-anxiety and HADS-depression were −0.67 and −0.53, respectively, while symptom burden correlated with HADS-anxiety at ρ = 0.50 and HADS-depression at ρ = 0.44 (all *p* < 0.001 where marked in Table 7).

### 3.8. Development and Internal Validation of the Risk Model

The multivariable logistic model for clinically significant distress and its internally validated performance are presented in Table 8, with discrimination illustrated in Figure 2 and calibration in Figure 3.

In the multivariable model, FACIT-Sp Peace (OR 0.72 per point, 95% CI 0.61 to 0.85; *p* < 0.001) and composite symptom burden (OR 1.37 per 10 points, 95% CI 1.11 to 1.69; *p* = 0.003) remained significant. The apparent AUC was 0.816 (95% CI 0.748 to 0.875) and the optimism-corrected AUC was 0.789; the corrected calibration slope was 0.863 and the corrected Brier score was 0.191.

The clinical model had an AUC of 0.752 (95% CI 0.679 to 0.825), the spiritual model 0.788 (95% CI 0.718 to 0.852), and the full model 0.816 (95% CI 0.748 to 0.875). The paired AUC difference between the full and clinical models was 0.064 (95% CI 0.008 to 0.12; *p* = 0.023). At the Youden-optimal threshold (0.471), sensitivity was 76.7% and specificity 75.3%.

Observed versus predicted risk was 11% versus 13% in the lowest decile, 35% versus 34% in the fourth, 65% versus 65% in the seventh and 94% versus 96% in the highest decile.

### 3.9. Sensitivity, Propensity-Score and Decision-Curve Analyses

The robustness of the profile–distress association and the effect of spiritual help-seeking were examined in eleven prespecified sensitivity and propensity-score analyses and, following the source-document verification (Section 2.3) and the missing-data sensitivity analyses (Section 2.4), in three further post hoc analyses (Table 9), and the clinical utility of the risk model was assessed by decision-curve analysis (Figure 4).

The primary adjusted odds ratio for the integrated profile was 0.21 (95% CI 0.10 to 0.45; *p* < 0.001) and ranged from 0.22 to 0.26 in the principal restriction and classification-sensitivity analyses. Re-estimating the profiles with the prorated RCI-10 totals again selected the three-profile solution (BIC 2180.9 versus 2185.7 for four profiles; entropy 0.845), with class sizes of 61, 53 and 61 and with 173 of 175 patients (98.9%; κ = 0.98) retaining their modal class; the adjusted odds ratio for the integrated profile versus the other profiles was 0.22 (95% CI 0.10 to 0.46), and the Class 3 versus Class 1 estimates for distress (OR 0.20, 95% CI 0.08 to 0.46), global quality of life (13.8 points, 95% CI 4.0 to 23.6) and composite symptom burden (−11.6 points, 95% CI −18.4 to −4.8) were essentially unchanged. Rescaling the FACIT-Sp subscales to the 0–16 metric left the profile assignment, the adjusted odds ratios and the AUC of the risk model identical, as expected for a linear transformation of standardised indicators. Under multiple imputation the adjusted odds ratio was 0.22 (95% CI 0.10 to 0.47) and the apparent AUC of the full model was 0.814 (clinical-only model 0.751); the nine patients (5.1%) with any missing value did not differ from the remainder in profile membership (*p* = 0.71) or distress status (*p* = 0.48). The IPTW estimate for spiritual help-seeking was 0.72 (95% CI 0.47 to 1.10; *p* = 0.125), and the propensity-score matched estimate was 0.88 (95% CI 0.32 to 2.38; *p* = 0.800).

Across threshold probabilities of 5–85%, the full model had net benefit at least as high as the clinical model. Net benefit for the full versus clinical model was 0.397 versus 0.394 at a 20% threshold, 0.301 versus 0.278 at 40%, and 0.203 versus 0.180 at 60%.

## 4. Discussion

### 4.1. Analysis of Findings

The three principal results of this study form a single line of argument. The latent profile analysis established that a clinically distinct high-risk group exists: patients who are religiously committed and observant yet lack inner peace and meaning, and whose prevalence of distress approaches that of patients with no spiritual resources at all. The prediction model then showed that this vulnerability can be captured in practice, because a brief measure of inner peace added discriminative and net-benefit value to routinely available clinical information, providing a screening tool with which such patients could be identified. Finally, the null association between spiritual help-seeking and distress indicates that these patients are not being reached by the default pathway in which support follows a request: seeking spiritual support was almost universal in the discordant profile, yet it was not accompanied by lower distress, so passively waiting for patients to ask does not address the needs of this group. Together the findings move from phenomenological discovery, to a means of identification, to the case for active rather than reactive assessment; the remainder of this section considers each element in turn.

The central finding is not simply that greater spirituality was associated with less distress, but that the configuration of spiritual resources mattered. Nearly one third of the cohort showed a discordant pattern: religious commitment, attendance and Faith were high, while Peace and Meaning remained comparatively low. Their distress prevalence (61.5%) was close to the low-resource profile (68.9%) and more than twice that of the integrated profile (25.8%). This extends person-centred work showing that combinations of religious characteristics can be more informative than single scale scores [22] and is consistent with oncology studies in which spiritual well-being profiles relate to psychosocial functioning [9]. It also accords with variable-centred evidence that the existential dimensions of spirituality, especially peace and meaning, are more closely related to psychological outcomes than faith or religious practice alone [23,24,25], and with recent cross-sectional studies in which peace was the dimension most consistently related to lower psychological symptoms [26] and spiritual well-being was inversely associated with both anxiety and depression [27].

The discordance between religious engagement and inner peace has an important conceptual implication. Religious involvement should not be assumed to indicate that spiritual needs are resolved. Religious coping can include supportive as well as conflict-laden forms; negative religious coping encompasses feelings of abandonment, punishment, anger toward God and unresolved spiritual struggle [28], and such struggle has been associated with adverse health outcomes [29]. Class 2 may therefore capture patients who remain behaviourally and cognitively religious while experiencing unresolved existential distress. This distinction may be especially visible in Romania, where religious affiliation and practice are culturally common [30], because high religiosity provides less discrimination between patients than variation in peace and meaning.

Clinically, the findings argue for spiritual assessment that moves beyond affiliation, service attendance and the stated importance of religion. Those indicators would make Classes 2 and 3 appear similar, yet the two groups differed markedly in distress and quality of life. Contemporary spiritual-care frameworks emphasise assessment of concerns, meaning, connectedness and spiritual suffering rather than religious identity alone [6,7]. Our results support that approach and suggest that a brief measure focused on inner peace may add useful information to routine distress screening. Patient-reported spiritual concerns may also help identify individuals who are receptive to chaplaincy or related support [31], and a single screening item asking whether religion or spirituality currently provides the strength and comfort a patient needs has recently shown acceptable sensitivity and specificity for religious or spiritual distress in outpatient oncology [32], but the present data indicate that clinicians should not wait for an explicit request for spiritual help before assessing need.

The prediction analysis reinforces the same message from a different methodological angle. Adding spiritual variables to routine clinical predictors increased the AUC from 0.752 to 0.816, with an optimism-corrected AUC of 0.789, and the full model showed positive incremental net benefit across clinically relevant thresholds. FACIT-Sp Peace provided the largest independent contribution, whereas Faith, ECOG status, help-seeking and sex were not independently significant once the complete predictor set was considered. Clinical and sociodemographic variables alone often have only modest performance for predicting distress in oncology populations [33]. The present model therefore suggests that a compact patient-reported existential measure can complement information already available in the oncology record. It should be viewed as a triage aid rather than a replacement for validated symptom screening: its potential value is to prioritise closer psychological or spiritual assessment when referral capacity is limited.

Internal validation is particularly important because prediction performance estimated in the development cohort is usually optimistic. The bootstrap-corrected calibration slope of 0.863 and the small reduction in AUC after optimism correction suggest some, but not extreme, overfitting. Calibration deserves emphasis because a model used to trigger referral must provide probabilities that are reasonably compatible with observed risk, not merely rank patients correctly [19]. The decision-curve results further indicate that the incremental discrimination translated into measurable net benefit across a range of referral thresholds [20]. Nevertheless, the model remains a development model and external validation is required before any threshold is adopted for clinical use; this is consistent with TRIPOD principles [17,18].

The null propensity-score result for spiritual help-seeking requires careful interpretation. Because help-seeking is self-selected, this finding should not be interpreted as evidence that spiritual care is ineffective; patients with greater need may be more likely to seek support, and the cross-sectional design cannot determine whether distress preceded or followed help-seeking. Instead, the result highlights the difference between receiving or requesting religious/spiritual support and achieving existential well-being. Prospective research should test whether the profiles are stable over time, whether transitions in Peace and Meaning track changes in distress, and whether patients with discordant profiles preferentially benefit from interventions that explicitly address meaning, dignity and existential concerns, such as meaning-centred psychotherapy or dignity therapy [34,35].

### 4.2. Study Limitations

Several limitations must be weighed. The design is cross-sectional, so the temporal ordering of spiritual configuration and distress cannot be established; it is entirely plausible that distress erodes inner peace rather than the reverse, or that both are consequences of a third process, and the language of protection used above should be read as descriptive rather than causal. Latent profile analysis is a model-based procedure, and although the three-profile solution was supported by the Bayesian information criterion, the bootstrap likelihood-ratio test, high entropy and stability after excluding uncertainly classified patients, latent profiles are statistical constructs whose replication in independent samples is essential before they are treated as real clinical types; with 175 patients the enumeration is necessarily provisional and a larger sample might resolve further subgroups. The prediction model was developed and internally validated in the same study cohort, and although optimism correction addresses overfitting it cannot substitute for external validation in a geographically and temporally distinct cohort; the reported performance should therefore be regarded as an upper bound on what would be observed elsewhere. The cohort was recruited at a single centre in one Romanian city and is culturally and religiously homogeneous, with more than four-fifths Orthodox, so the near-constancy of religious commitment that makes the discordant profile visible here may not obtain in religiously plural settings. The instrument battery was locally adapted, and the deviations bear directly on the profile-defining scales: the FACIT-Sp-12 was administered on a four-point rather than the validated five-point response format, so subscale scores range from 4 to 16 rather than 0 to 16 and are not directly comparable with published norms, and eleven RCI-10 totals had originally been entered as raw sums over incomplete item sets and fell below the nominal minimum of the scale. The source-document verification described in Section 2.3 established the origin of both deviations, recovered the complete RCI-10 item-level data, and showed that rescoring the affected totals left the profile solution and the profile–distress associations unchanged (Section 3.9). Several EORTC QLQ-C30 items also used a non-standard response format, and three HADS items required re-keying, which was confirmed against the original questionnaires. Because the profiles are defined by relative position on these indicators rather than by absolute scores the enumeration is unlikely to be distorted, but the absolute values reported here should not be transported to other cohorts. HADS thresholds identify probable rather than diagnosed cases, so the outcome is screen-detected distress rather than psychiatric diagnosis. A conceptual limitation also deserves emphasis. The study identified a discordant profile, but it did not measure negative religious coping or spiritual struggle directly; the FACIT-Sp and RCI-10 capture the presence of peace, meaning and religious commitment rather than feelings of abandonment, punishment or conflict with God. It therefore remains unclear whether patients in Class 2 experience genuine spiritual struggle, are religiously engaged in ways that do not translate into existential comfort, or are simply reporting the low peace that accompanies a high symptom burden, and the mechanistic interpretation offered above is a hypothesis rather than a demonstrated pathway. Future studies should incorporate a validated measure of negative religious coping, such as the negative religious coping subscale of the RCOPE [28], alongside the profile indicators, in order to test whether the discordant configuration is characterised by spiritual struggle and to validate it as a clinical subtype. Unmeasured confounders including social support, financial strain, personality and prior psychiatric history were unavailable, the propensity-score matched analysis retained only 33 pairs and is correspondingly underpowered, and no multiplicity correction was applied to the exploratory analyses.

## 5. Conclusions

A person-centred analysis of spirituality in patients with cancer identified three distinct configurations and, critically, showed that religious commitment and existential well-being can diverge within the same patient. Those who were religiously engaged but lacked inner peace carried a burden of psychological distress as heavy as patients with no spiritual resources at all, while only the integrated configuration was associated with substantially lower distress and better quality of life; because the design is cross-sectional these are associations, not demonstrated effects. Because conventional spiritual screening records affiliation and religious practice, it would classify these two very differently positioned groups as equivalent, and the act of seeking spiritual support proved to be a marker of religious engagement rather than an indicator of need being met. Incorporating a brief measure of inner peace into distress risk assessment improved discrimination and clinical utility beyond routine clinical variables. Prospective, externally validated studies in religiously diverse populations are now required.

## Figures and Tables

**Figure 1:**
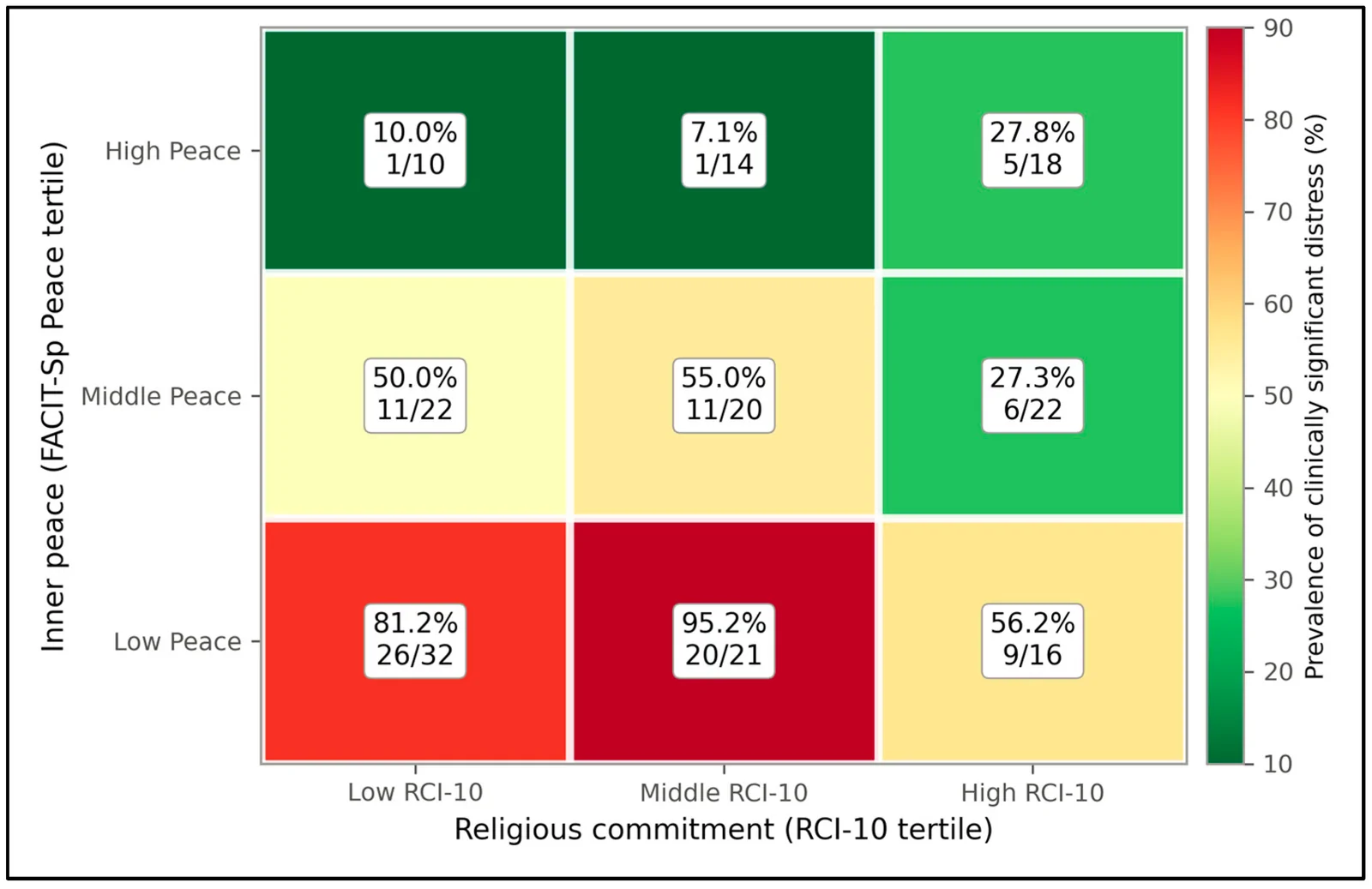
Observed prevalence of clinically significant psychological distress (HADS-anxiety or HADS-depression ≥8) across the joint cross-classification of FACIT-Sp Peace tertile and Religious Commitment Inventory-10 tertile. Each cell displays the prevalence and the number of events over the number of patients. Abbreviations: FACIT-Sp, Functional Assessment of Chronic Illness Therapy—Spiritual Well-Being; HADS, Hospital Anxiety and Depression Scale; RCI-10, Religious Commitment Inventory-10.

**Figure 2:**
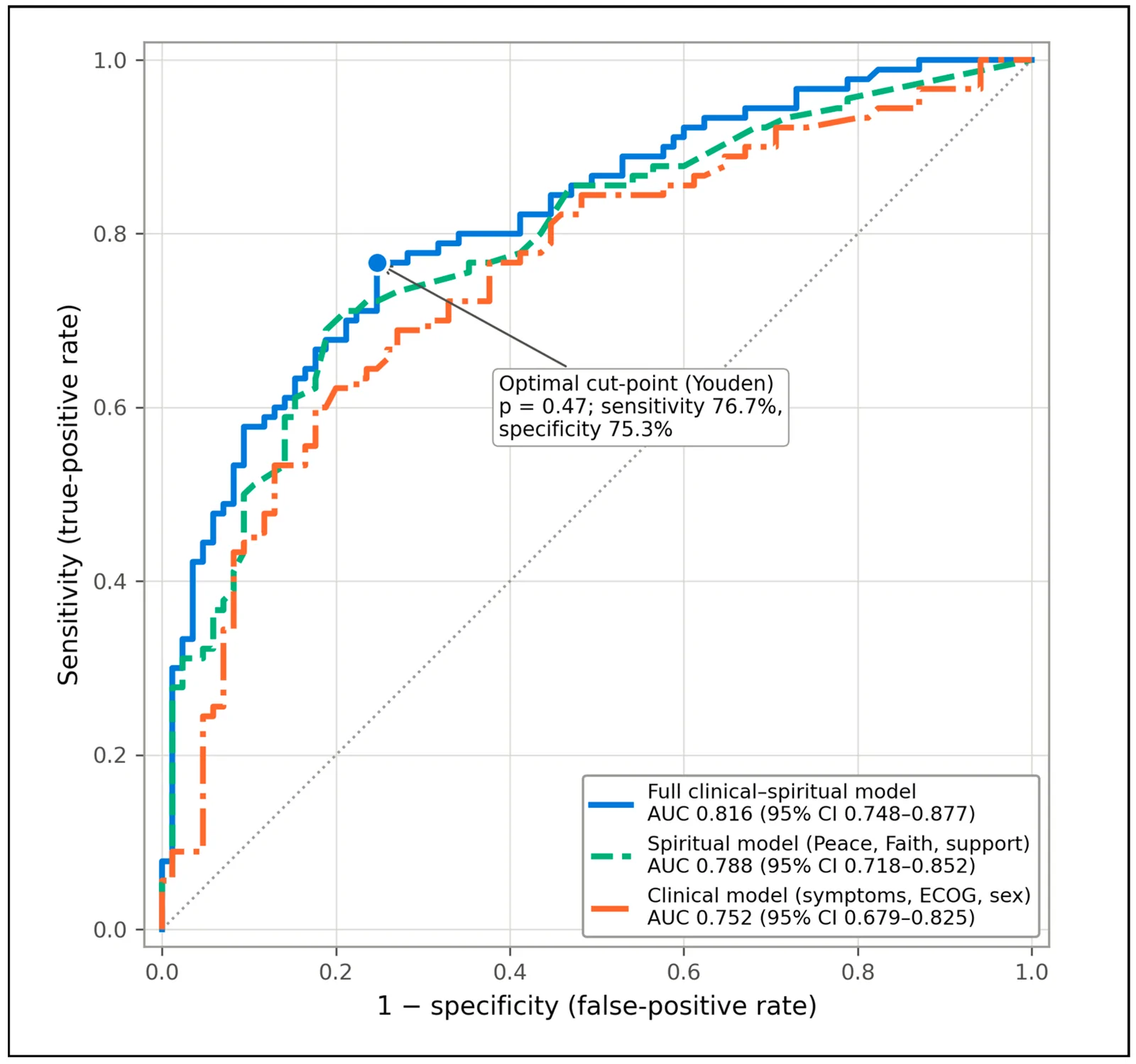
Receiver operating characteristic curves for the full clinical–spiritual model, the spiritual-only model and the clinical-only model for the prediction of clinically significant psychological distress, with bootstrap confidence intervals for the area under the curve and the Youden-optimal operating point of the full model. Abbreviations: AUC, area under the curve; ECOG, Eastern Cooperative Oncology Group.

**Figure 3:**
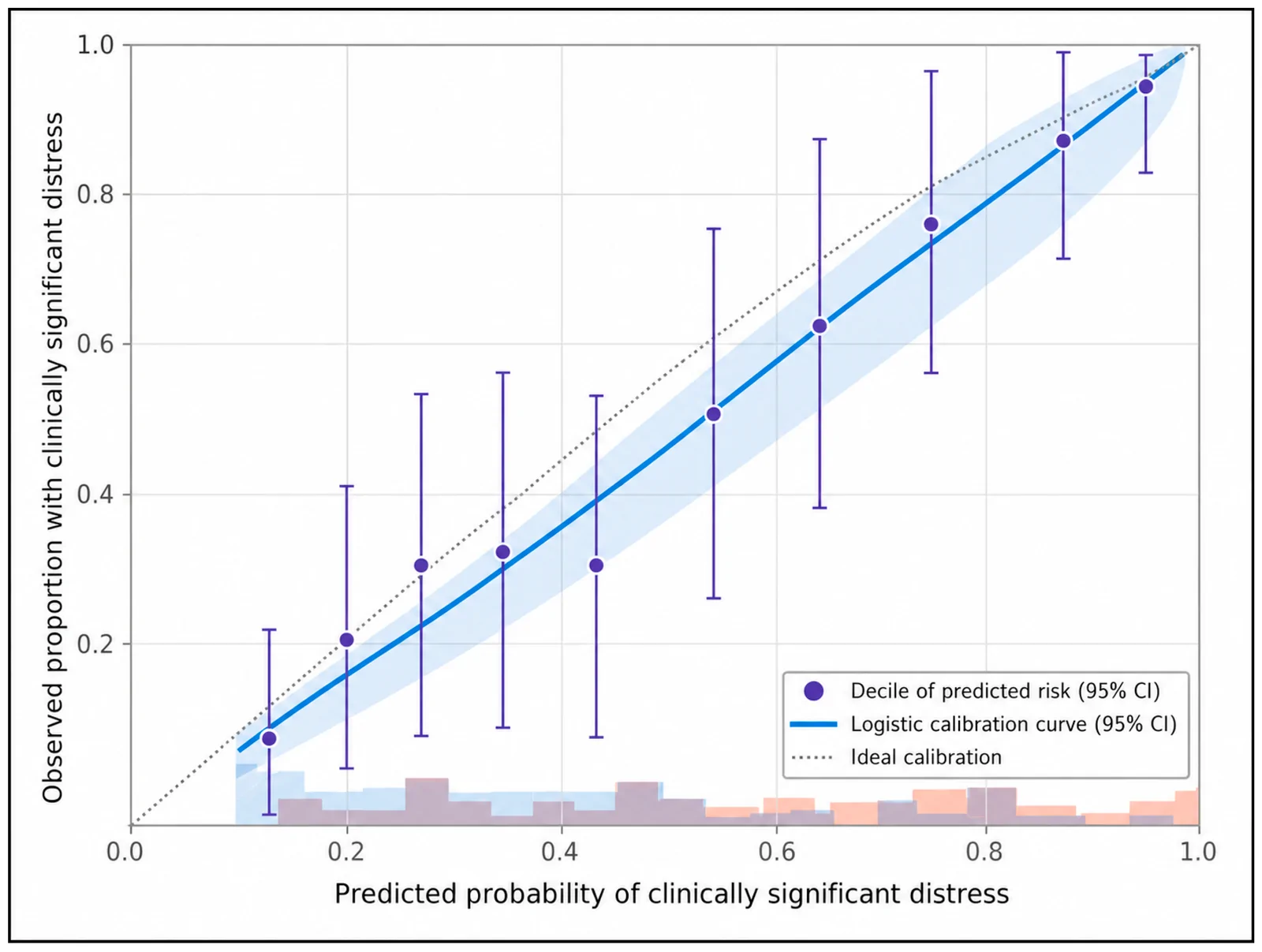
Calibration of the full clinical–spiritual model. Points show observed versus mean predicted risk by decile of predicted probability with 95% confidence intervals; the solid line is the logistic calibration curve with its confidence band; the dotted diagonal is ideal calibration. Histograms at the base show the distribution of predicted probabilities among patients with and without distress.

**Figure 4:**
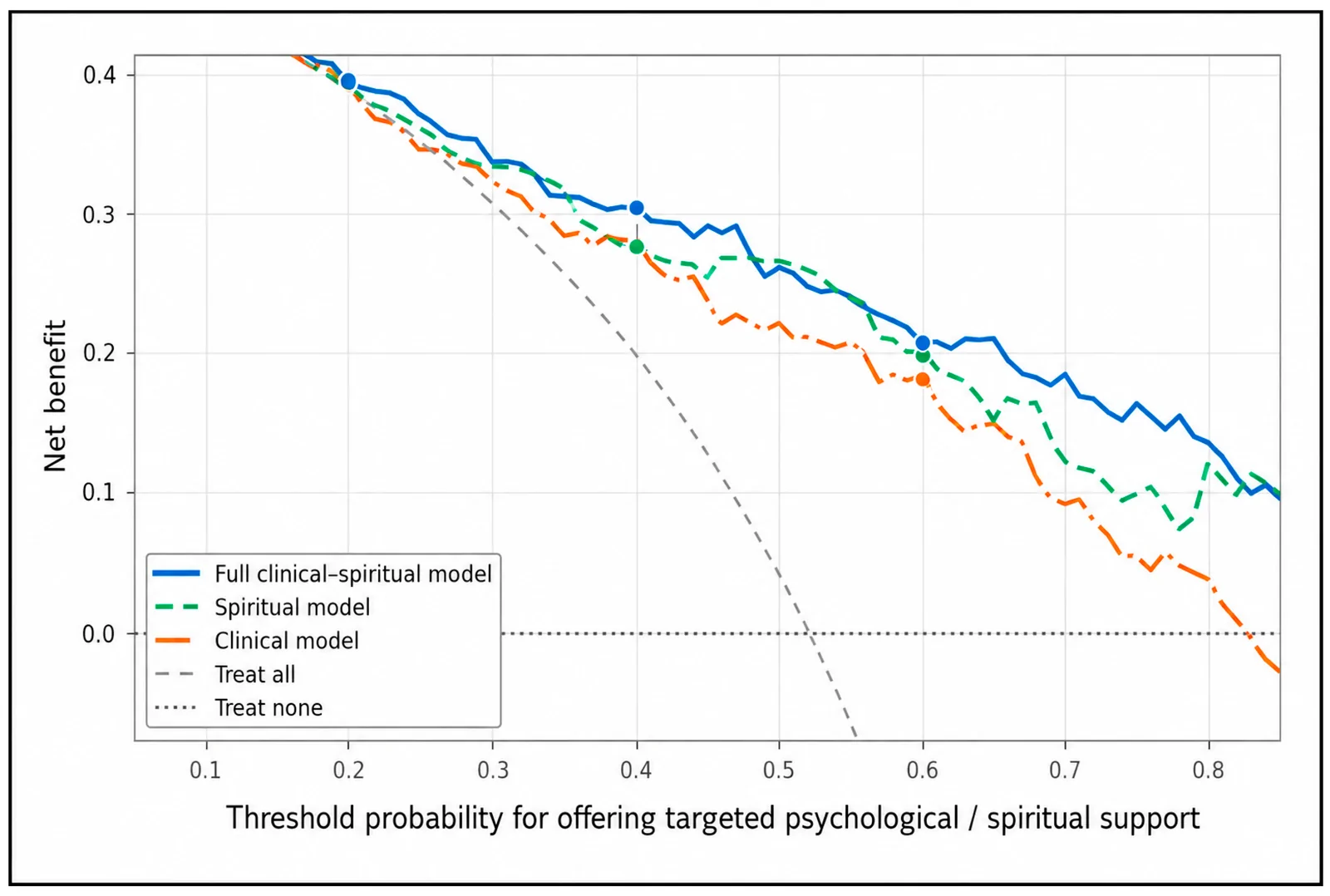
Decision-curve analysis showing the net benefit of the full clinical–spiritual model, the spiritual-only model and the clinical-only model relative to the default strategies of offering targeted psychological or spiritual support to all patients or to none, across threshold probabilities from 5% to 85%.

**Table 1:** Model-fit statistics for latent profile solutions with one to five profiles.

Number of Profiles	Log-Likelihood	Free Parameters	AIC	BIC	SABIC	Entropy	Smallest Class (%)	Class Sizes	BLRT Statistic	*p*, BLRT ^a^
1	−1239.6	10	2499.2	2530.8	2499.1	–	100.0	175	–	–
2	−1080.5	21	2203.0	2269.4	2202.9	0.855	36.0	112/63	318.2	0.005
3	−1007.2	32	2078.3	2179.6	2078.3	0.847	29.7	62/61/52	146.7	0.005
4	−981.0	43	2048.1	2184.2	2048.0	0.849	12.0	60/51/43/21	52.2	0.005
5	−952.9	54	2013.7	2184.6	2013.6	0.857	12.0	43/40/38/33/21	56.4	0.005

Models are Gaussian finite mixtures with diagonal covariance fitted to five standardised indicators (FACIT-Sp Meaning, Peace and Faith, RCI-10 total and frequency of service attendance), with 100 random starts per solution. ^a^ Parametric bootstrap likelihood-ratio test of the k-profile against the (k − 1)-profile solution, 200 replicates; the minimum attainable *p* value is 0.005. Abbreviations: AIC, Akaike information criterion; BIC, Bayesian information criterion; BLRT, bootstrap likelihood-ratio test; FACIT-Sp, Functional Assessment of Chronic Illness Therapy—Spiritual Well-Being; RCI-10, Religious Commitment Inventory-10; SABIC, sample-size-adjusted Bayesian information criterion.

**Table 2:** Profile-defining indicators and illness-related spirituality variables by latent profile.

Indicator	Class 1 (*n* = 61)	Class 2 (*n* = 52)	Class 3 (*n* = 62)	Total (*n* = 175)	Standardised Means (Class 1/2/3)	F	*p* Value	Effect Size
FACIT-Sp Meaning (4–16)	12.2 ± 3.0	13.5 ± 1.7	15.9 ± 0.3	13.9 ± 2.5	−0.65/−0.17/+0.78	51.5	<0.001	0.375
FACIT-Sp Peace (4–16)	11.0 ± 3.4	11.6 ± 2.0	15.3 ± 1.0	12.7 ± 3.0	−0.54/−0.37/+0.84	57.0	<0.001	0.399
FACIT-Sp Faith (4–16)	9.4 ± 2.9	14.3 ± 2.0	15.1 ± 1.4	12.9 ± 3.4	−1.03/+0.43/+0.66	119.9	<0.001	0.582
RCI-10 total (8–46)	14.9 ± 5.1	31.1 ± 5.8	30.1 ± 8.3	25.1 ± 10.0	−1.03/+0.60/+0.50	113.5	<0.001	0.569
Frequency of service attendance (1–6)	2.0 ± 1.0	3.9 ± 1.2	3.7 ± 1.1	3.2 ± 1.4	−0.85/+0.54/+0.39	56.3	<0.001	0.395
Sought spiritual support, *n* (%)	25 (41.0)	47 (90.4)	55 (88.7)	127 (72.6)	–	–	<0.001	0.518
Religious practice intensified, *n* (%)	18 (29.5)	31 (59.6)	39 (62.9)	88 (50.3)	–	–	<0.001	0.305
Beliefs changed positively, *n* (%)	53 (86.9)	51 (98.1)	62 (100.0)	166 (94.9)	–	–	0.002	0.266

Class 1, low spiritual–religious resource; Class 2, religiously engaged but existentially unsettled; Class 3, integrated spiritual–religious resource. Data are mean ± standard deviation or *n* (%). Standardised means are expressed in sample standard-deviation units. Effect sizes are η^2^ for continuous and Cramér’s V for categorical variables. The final three rows were not used to estimate the profiles. Abbreviations: FACIT-Sp, Functional Assessment of Chronic Illness Therapy—Spiritual Well-Being; RCI-10, Religious Commitment Inventory-10.

**Table 3:** Demographic and clinical characteristics of the study population by latent spiritual–religious profile.

Characteristic	Class 1 (*n* = 61)	Class 2 (*n* = 52)	Class 3 (*n* = 62)	Total (*n* = 175)	*p* Value	Effect Size
Age, years	60.8 ± 13.2	62.4 ± 13.1	63.3 ± 13.2	62.2 ± 13.1	0.580	0.006
Sex, *n* (%)						
Female	22 (36.1)	27 (51.9)	40 (64.5)	89 (50.9)	0.007	0.239
Male	39 (63.9)	25 (48.1)	22 (35.5)	86 (49.1)		
Residence, *n* (%)						
Urban	41 (67.2)	22 (42.3)	37 (59.7)	100 (57.1)	0.025	0.205
Rural	20 (32.8)	30 (57.7)	25 (40.3)	75 (42.9)		
Education, *n* (%)						
Lower secondary	6 (9.8)	15 (28.8)	12 (19.4)	33 (18.9)	0.109	0.172
Vocational	15 (24.6)	12 (23.1)	9 (14.5)	36 (20.6)		
High school	29 (47.5)	16 (30.8)	25 (40.3)	70 (40.0)		
University	11 (18.0)	9 (17.3)	16 (25.8)	36 (20.6)		
Marital status, *n* (%)						
Married	37 (60.7)	32 (61.5)	35 (56.5)	104 (59.4)	0.168	0.161
Widowed	7 (11.5)	12 (23.1)	16 (25.8)	35 (20.0)		
Single	11 (18.0)	6 (11.5)	4 (6.5)	21 (12.0)		
Divorced	6 (9.8)	2 (3.8)	7 (11.3)	15 (8.6)		
Primary tumour site, *n* (%)						
Colorectal	15 (24.6)	15 (28.8)	14 (22.6)	44 (25.1)	0.892	0.136
Lung	7 (11.5)	10 (19.2)	13 (21.0)	30 (17.1)		
Breast	11 (18.0)	7 (13.5)	11 (17.7)	29 (16.6)		
Cervical/uterine	1 (1.6)	3 (5.8)	2 (3.2)	6 (3.4)		
Prostate	1 (1.6)	1 (1.9)	1 (1.6)	3 (1.7)		
Bladder	1 (1.6)	1 (1.9)	0 (0.0)	2 (1.1)		
Other	25 (41.0)	15 (28.8)	21 (33.9)	61 (34.9)		
Disease status, *n* (%)						
Localised	17 (27.9)	14 (26.9)	19 (30.6)	50 (28.6)	0.474	0.126
Metastatic	35 (57.4)	30 (57.7)	38 (61.3)	103 (58.9)		
Terminal	3 (4.9)	6 (11.5)	2 (3.2)	11 (6.3)		
Remission	6 (9.8)	2 (3.8)	3 (4.8)	11 (6.3)		
Time since diagnosis, months	24.5 (15.5–47.2)	26.0 (17.8–69.8)	24.0 (15.5–66.8)	25.0 (16.0–58.0)	0.673	0.014
ECOG performance status	1.0 (1.0–2.0)	1.0 (0.0–2.0)	1.0 (0.0–1.0)	1.0 (0.0–2.0)	0.009	0.060
Comorbidities, *n*	2.0 (1.0–3.0)	2.0 (1.0–3.2)	2.0 (1.2–3.0)	2.0 (1.0–3.0)	0.615	0.003
Chemotherapy, *n* (%)						
Yes	43 (70.5)	43 (82.7)	44 (71.0)	130 (74.3)	0.254	0.125
No	18 (29.5)	9 (17.3)	18 (29.0)	45 (25.7)		
Surgery, *n* (%)						
Yes	22 (36.1)	19 (36.5)	17 (27.4)	58 (33.1)	0.491	0.090
No	39 (63.9)	33 (63.5)	45 (72.6)	117 (66.9)		
Religious affiliation, *n* (%)						
Orthodox	53 (86.9)	43 (82.7)	46 (74.2)	142 (81.1)	0.194	0.157
Catholic	6 (9.8)	4 (7.7)	7 (11.3)	17 (9.7)		
Protestant/Neo-Protestant	2 (3.3)	5 (9.6)	6 (9.7)	13 (7.4)		
Other	0 (0.0)	0 (0.0)	3 (4.8)	3 (1.7)		

Class 1, low spiritual–religious resource; Class 2, religiously engaged but existentially unsettled; Class 3, integrated spiritual–religious resource. Data are mean ± standard deviation, median (interquartile range) or *n* (%). Effect sizes are η^2^ for continuous and Cramér’s V for categorical variables. Abbreviations: ECOG, Eastern Cooperative Oncology Group.

**Table 4:** Psychological distress and quality of life outcomes by latent spiritual–religious profile.

Outcome	Class 1 (*n* = 61)	Class 2 (*n* = 52)	Class 3 (*n* = 62)	*p* Value	Effect Size	Adjusted Class 3 vs. Class 1 (95% CI) ^a^	*p*, Adjusted
HADS-Anxiety score	7.3 ± 5.5	6.8 ± 5.0	3.0 ± 2.6	<0.001	0.167	−3.9 (−5.6 to −2.3)	<0.001
HADS-Depression score	9.6 ± 5.1	9.4 ± 4.4	5.8 ± 3.9	<0.001	0.132	−3.2 (−4.9 to −1.4)	<0.001
HADS total score	16.9 ± 9.2	16.2 ± 8.3	8.8 ± 5.4	<0.001	0.190	−7.1 (−10.0 to −4.3)	<0.001
Global health status/QoL	57.1 ± 28.3	59.0 ± 23.6	75.8 ± 20.8	<0.001	0.111	14.1 (4.4 to 23.8)	0.004
Functional summary score	59.1 ± 24.7	59.2 ± 23.9	75.5 ± 15.4	<0.001	0.117	10.7 (3.2 to 18.3)	0.005
Emotional functioning	65.6 ± 31.5	62.8 ± 28.8	85.1 ± 17.1	<0.001	0.128	15.4 (5.4 to 25.4)	0.003
Social functioning	56.3 ± 38.0	59.3 ± 36.7	69.1 ± 31.2	0.115	0.025	4.0 (−9.1 to 17.0)	0.552
Composite symptom burden	33.3 ± 23.3	34.6 ± 23.3	18.4 ± 14.4	<0.001	0.117	−11.8 (−18.6 to −5.0)	<0.001
Clinically significant distress (HADS-A or HADS-D ≥ 8), *n* (%)	42 (68.9)	32 (61.5)	16 (25.8)	<0.001	0.384	0.19 (0.08 to 0.44) ^†^	<0.001
Clinically significant anxiety (HADS-A ≥ 8), *n* (%)	27 (44.3)	20 (38.5)	3 (4.8)	<0.001	0.393	0.07 (0.02 to 0.27) ^†^	<0.001
Clinically significant depression (HADS-D ≥ 8), *n* (%)	39 (63.9)	31 (59.6)	15 (24.2)	<0.001	0.363	0.21 (0.09 to 0.50) ^†^	<0.001
Probable case (HADS-A or HADS-D ≥ 11), *n* (%)	31 (50.8)	25 (48.1)	7 (11.3)	<0.001	0.382	0.12 (0.04 to 0.33) ^†^	<0.001

Class 1, low spiritual–religious resource; Class 2, religiously engaged but existentially unsettled; Class 3, integrated spiritual–religious resource. Data are mean ± standard deviation or *n* (%). ^a^ Adjusted for age, sex, advanced disease, ECOG performance status and comorbidity count; values are mean differences from linear models with HC3 standard errors except where marked ^†^, which are odds ratios from logistic models. Abbreviations: CI, confidence interval; ECOG, Eastern Cooperative Oncology Group; HADS, Hospital Anxiety and Depression Scale; HADS-A, HADS anxiety subscale; HADS-D, HADS depression subscale; QoL, quality of life.

**Table 5:** Subgroup analyses of the association between the integrated spiritual–religious profile and clinically significant psychological distress.

Subgroup	*n*	Events, *n* (%)	Adjusted OR, Class 3 vs. Classes 1–2 (95% CI) ^a^	*p* Value	*p* for Interaction
Overall	175	90 (51.4)	0.22 (0.10 to 0.44)	<0.001	
Female	89	42 (47.2)	0.16 (0.06 to 0.41)	<0.001	0.347
Male	86	48 (55.8)	0.33 (0.11 to 0.96)	0.043	0.347
Age < 65 years	75	39 (52.0)	0.18 (0.05 to 0.60)	0.005	0.685
Age ≥ 65 years	100	51 (51.0)	0.24 (0.09 to 0.60)	0.002	0.685
Advanced disease	114	63 (55.3)	0.25 (0.11 to 0.62)	0.002	0.596
Non-advanced disease	61	27 (44.3)	0.16 (0.04 to 0.59)	0.006	0.596
ECOG 0–1	113	46 (40.7)	0.26 (0.11 to 0.61)	0.002	0.359
ECOG 2–4	62	44 (71.0)	0.12 (0.03 to 0.54)	0.006	0.359
Urban residence	100	47 (47.0)	0.22 (0.08 to 0.58)	0.002	0.778
Rural residence	75	43 (57.3)	0.19 (0.06 to 0.58)	0.004	0.778
Symptom burden below median	87	30 (34.5)	0.29 (0.10 to 0.79)	0.016	0.565
Symptom burden at/above median	88	60 (68.2)	0.20 (0.06 to 0.65)	0.007	0.565

^a^ Odds ratios from logistic regression adjusted for age, sex, advanced disease and ECOG performance status; in subgroups where a covariate was constant or quasi-separation occurred, the covariate set was reduced in a prespecified order. *p* for interaction is from the multiplicative interaction term in the corresponding full-cohort model. Abbreviations: CI, confidence interval; ECOG, Eastern Cooperative Oncology Group; OR, odds ratio.

**Table 6:** Univariable logistic associations of candidate predictors with clinically significant psychological distress.

Candidate Predictor	Events/*n*	Odds Ratio	95% CI	*p* Value	AUC Alone
FACIT-Sp Peace (per +1 point)	90/175	0.65	0.56 to 0.76	<0.001	0.784
FACIT-Sp Meaning (per +1 point)	90/175	0.71	0.61 to 0.83	<0.001	0.697
FACIT-Sp Faith (per +1 point)	90/175	0.84	0.76 to 0.93	<0.001	0.640
RCI-10 total (per +1 point)	90/175	0.95	0.93 to 0.99	0.004	0.629
Service attendance at least monthly	90/175	0.48	0.26 to 0.90	0.021	0.585
Sought spiritual support during illness	90/175	0.54	0.27 to 1.06	0.074	0.561
Composite symptom burden (per +10 points)	90/175	1.56	1.30 to 1.87	<0.001	0.743
ECOG (per +1 category)	90/175	1.59	1.20 to 2.11	0.001	0.643
Age (per +10 years)	90/175	0.93	0.74 to 1.17	0.557	0.524
Male sex	90/175	1.41	0.78 to 2.56	0.254	0.543
Advanced disease	90/175	1.56	0.83 to 2.91	0.166	0.550
Urban residence	90/175	0.66	0.36 to 1.21	0.177	0.551
Comorbidity count (per +1)	90/175	1.03	0.84 to 1.26	0.796	0.501
Active chemotherapy	90/175	0.90	0.46 to 1.78	0.767	0.510
Time since diagnosis (per +12 months)	86/168	0.96	0.89 to 1.04	0.344	0.544

Odds ratios are per unit indicated. AUC alone is the area under the receiver operating characteristic curve for a model containing that predictor only. Abbreviations: AUC, area under the curve; CI, confidence interval; ECOG, Eastern Cooperative Oncology Group; FACIT-Sp, Functional Assessment of Chronic Illness Therapy—Spiritual Well-Being; RCI-10, Religious Commitment Inventory-10.

**Table 7:** Spearman rank correlation matrix of spirituality predictors, clinical variables and outcomes.

Variable	1	2	3	4	5	6	7	8	9	10
1. Peace	1									
2. Meaning	0.63 ***	1								
3. Faith	0.39 ***	0.42 ***	1							
4. RCI-10	0.26 ***	0.35 ***	0.70 ***	1						
5. Attendance	0.15	0.25**	0.47 ***	0.55 ***	1					
6. Symptom burden	−0.46 ***	−0.43 ***	−0.15 *	−0.17 *	−0.07	1				
7. ECOG	−0.34 ***	−0.33 ***	−0.17 *	−0.19 *	−0.10	0.50 ***	1			
8. HADS-A	−0.67 ***	−0.42 ***	−0.26 ***	−0.18 *	−0.07	0.50 ***	0.32 ***	1		
9. HADS-D	−0.53 ***	−0.42 ***	−0.23 **	−0.18 *	−0.10	0.44 ***	0.32 ***	0.60 ***	1	
10. Global QoL	0.40 ***	0.40 ***	0.20 **	0.21 **	0.15 *	−0.59 ***	−0.46 ***	−0.43 ***	−0.43 ***	1

Values are Spearman’s ρ. * *p* < 0.05; ** *p* < 0.01; *** *p* < 0.001. Column numbers correspond to the numbered row variables. Abbreviations: ECOG, Eastern Cooperative Oncology Group; HADS-A, Hospital Anxiety and Depression Scale—anxiety; HADS-D, Hospital Anxiety and Depression Scale—depression; QoL, quality of life; RCI-10, Religious Commitment Inventory-10.

**Table 8:** Multivariable logistic model for clinically significant psychological distress with internally validated performance measures.

Predictor	β	Odds Ratio	95% CI	Wald χ^2^	*p* Value
FACIT-Sp Peace (per +1 point)	−0.331	0.72	0.61 to 0.85	14.9	<0.001
FACIT-Sp Faith (per +1 point)	−0.040	0.96	0.83 to 1.12	0.3	0.607
Composite symptom burden (per +10 points)	0.315	1.37	1.11 to 1.69	8.6	0.003
ECOG (per +1 category)	0.001	1.00	0.70 to 1.43	0.0	0.998
Sought spiritual support during illness	−0.502	0.61	0.21 to 1.72	0.9	0.347
Male sex	0.178	1.19	0.57 to 2.50	0.2	0.637
**Model Performance**					
Area under the ROC curve (apparent)		0.816	0.748 to 0.875		
Area under the ROC curve (optimism-corrected)		0.789			
Calibration slope (apparent/corrected)		1.000/0.863			
Calibration-in-the-large		0.000			
Brier score (apparent/corrected)		0.174/0.191			
Nagelkerke R^2^		0.386			
Hosmer–Lemeshow test		χ^2^_8_ = 1.5			0.993
Likelihood-ratio test vs. null model					<0.001

Model developed in *n* = 175 patients with 90 events (15.0 events per candidate predictor); Akaike information criterion 196.6. Optimism correction by the Harrell bootstrap procedure with 500 resamples. The confidence interval for the apparent area under the curve is from 1000 bootstrap resamples. Abbreviations: CI, confidence interval; ECOG, Eastern Cooperative Oncology Group; FACIT-Sp, Functional Assessment of Chronic Illness Therapy—Spiritual Well-Being; ROC, receiver operating characteristic.

**Table 9:** Sensitivity and propensity-score analyses of the association between the integrated spiritual–religious profile, spiritual help-seeking and clinically significant distress.

Analysis	*n*	Events	Odds Ratio	95% CI	*p* Value
Primary analysis (Class 3 vs. Classes 1–2, adjusted)	175	90	0.21	0.10 to 0.45	<0.001
Excluding patients in remission	164	86	0.22	0.10 to 0.46	<0.001
Restricted to advanced disease	114	63	0.23	0.09 to 0.59	0.002
Restricted to ECOG 0–2	148	71	0.24	0.11 to 0.52	<0.001
Excluding uncertain class assignment (posterior <0.80)	153	77	0.26	0.12 to 0.56	<0.001
Alternative outcome: HADS-A ≥8 only	175	50	0.08	0.02 to 0.28	<0.001
Alternative outcome: HADS-D ≥8 only	175	85	0.22	0.11 to 0.47	<0.001
Alternative outcome: either subscale ≥11	175	63	0.13	0.05 to 0.32	<0.001
Additionally adjusted for symptom burden, education and tumour site	175	90	0.23	0.10 to 0.53	<0.001
Penalised (ridge) logistic regression	175	90	0.25	–	–
Verified data with prorated RCI-10 totals (profiles re-estimated) ^b^	175	90	0.22	0.10 to 0.46	<0.001
FACIT-Sp subscales rescaled to the 0–16 metric ^b^	175	90	0.21	0.10 to 0.45	<0.001
Multiple imputation of missing values (20 imputations) ^b^	175	90	0.22	0.10 to 0.47	<0.001
IPTW estimate for seeking spiritual support (outcome: distress)	175	90	0.72	0.47 to 1.10	0.125
1:1 propensity-score matched analysis (33 pairs, calliper 0.2 SD)	66	41	0.88	0.32 to 2.38	0.800

Unless otherwise stated, models are adjusted for age, sex, advanced disease, ECOG performance status and comorbidity count. The final two rows estimate the effect of seeking spiritual support rather than of profile membership. ^b^ Post hoc analyses added after source-document verification of the psychometric data (Section 2.3) and the missing-data sensitivity analyses (Section 2.4); in the prorated-RCI-10 analysis the profiles were re-estimated and 173 of 175 patients retained their modal class. Abbreviations: CI, confidence interval; ECOG, Eastern Cooperative Oncology Group; IPTW, inverse probability of treatment weighting.

## Data Availability

The data presented in this study are available on reasonable request from the corresponding author. The data are not publicly available because they contain information that could compromise the privacy of research participants.
